# Evaluation of HIV antigen /antibody combination ELISAs for diagnosis of HIV infection in Dar Es Salaam, Tanzania

**DOI:** 10.11604/pamj.2015.20.196.4934

**Published:** 2015-03-03

**Authors:** Loveness John Urio, Mohamed Ally Mohamed, Janneth Mghamba, Ahmed Abade, Said Aboud

**Affiliations:** 1 Tanzania Field Epidemiology and Laboratory Training Program, Tanzania; 2 Ministry of Health and Social Welfare, Dar Es Salaam, Tanzania; 3 Mirobiology and Immunology, Muhimbili University of Health and Allied Sciences, Ho, Ghana

**Keywords:** HIV antigen/antibody combination ELISA′s, HIV infection, diagnosis

## Abstract

**Introduction:**

The aim of this study was to evaluate the performance of Enzygnost HIV Integral II antigen/antibody combination ELISAs in order to formulate HIV ELISA testing algorithms for the Ministry of Health and Social Welfare, Tanzania.

**Methods:**

This was a laboratory-based evaluation of Enzygnost HIV Integral II Antibody/ Antigen, Murex HIV antigen/antibody and Vironostika HIV Uniform II antigen/antibody conducted between October 2011 and May 2012.

**Results:**

A total of 600 blood samples were included in the evaluation. A total of 209/596 (35.1%) serum samples were confirmed HIV positive. Of these, the prevalence of HIV infection was 2.3% (3/130), 2.3% (3/127), 2.2% (3/139) and 100% (200/200) for VCT clients, ANC attendees, blood donors and CTC patients, respectively. Three hundred and eighty seven (64.9%) were HIV negative samples. Sensitivity was 100% (95% CI; 98.3-100%) for all the three HIV ELISAs. The specificity for the Enzygnost HIV Integral II and Murex was 100% (95% CI; 99.1-100%). The final specificity at repeat testing was 99.5% (95% CI; 98.2-99.9%) for Vironostika. Enzygnost HIV Integral II detected HIV infection seven days since first bleed.

**Conclusion:**

Initial testing using either Vironostika or Murex HIV antigen/antibody combination ELISA followed by testing of reactive samples on the Enzygnost HIV Integral II gave a sensitivity and specificity of 100% with reduced window period. Combination of two HIV antigen/antibody combination ELISAs can be used as an alternative confirmatory testing strategy for screening of donated blood at the National and Zonal blood transfusion centres and in lab diagnosis of HIV infection.

## Introduction

Human immunodeficiency virus (HIV) infection remains an important public health concern in most of the developing countries including Tanzania. Early diagnosis of this infection is critical in providing effective antiviral treatment and to prevent transmission. One of the key issues to achieve this goal is to shorten the so-called “diagnostic window period” when the humoral immune response toward the virus is not fully developed during the acute phase of HIV-1 infection. The enzyme-linked immunosorbent assays (ELISAs) are the most commonly used techniques for the laboratory diagnosis of HIV infection [1]. The standard procedure for laboratory diagnosis of HIV infection usually includes screening for virus-specific antibodies using an enzyme-linked immunosorbent assay (ELISA) [2], followed by confirmatory testing of screening positive sample [1]. The UNAIDS/WHO has given recommendations for the use of combined screening assays for the diagnosis and confirmation of HIV infection [3]. Currently, a combination of antibody ELISA [4–7], or simple rapid assays [1] based on different test principles have been used in alternative diagnostic testing strategies for laboratory diagnosis in resource-limited countries. In developed countries, screening of HIV antibodies was followed by confirmatory testing, most commonly by using Western blot (WB) assay. However, testing strategy using WB is very costly for use in resource-limited countries like Tanzania [2, 8].

Recently, new fourth generation screening assays which permit a simultaneous detection of HIV antigen and antibody have been developed and have reduced the diagnostic window phase between time of HIV infection and laboratory diagnosis on average by four days in comparison to third-generation antibody assays because antibodies to HIV are absent in the very early phase of HIV infection [9–18]. With the p24 antigen combined in assays, HIV infections can be detected from few days to many weeks before antibody seroconversion, and this is useful in routine screening of blood [10, 15, 19]. The combined assays offer potential benefits of early detection of HIV infection via p24 Ag detection and also improve seroconversion sensitivity at a technical burden and financial cost [10, 15, 19, 20].

Evaluation of the assay is required once a new assay is introduced into use. The aim of the current study was to evaluate the performance and operational characteristics of HIV Antigen and Antibody Combination ELISAs for laboratory diagnosis of HIV infection in Dar es Salaam, Tanzania; and as well as to formulate HIV ELISA testing algorithms that could be used as an alternative HIV testing strategy for lab diagnosis of HIV infection.

## Methods

The study was a cross-sectional study design of blood samples conducted between October 2011 and January 2012. Altogether 600 serum samples were analysed (140 from National Blood Transfusion Centre (Eastern Zone Blood Transfusion Services); 130 from Voluntary Testing and Counselling (VCT); 130 from Antenatal Clinic (ANC) and 200 from Muhimbili- Care and Treatment Centre (CTC). In the evaluation, Enzygnost HIV Integral II Antibody/ Antigen (Siemens, Germany), Murex HIV antigen/antibody (Abbott Murex, UK) and Vironostika HIV Uniform II antigen/antibody (Biomerieux, the Netherlands) were included. INNO-LIA HIV I/II immunoblot (Innogenetics, Belgium) was used as a confirmatory test. All the three antigen/antibody combination HIV ELISA were evaluated for operational aspects such as storage conditions (2-8°C), incubation temperature (37°C), and need of special equipment, number of sera per run, price per kit and the performance appraisal of the assays on technical aspects using 10 technicians that performed the testing assays. These assessments, along with other selected assay characteristics, contributed to an overall appraisal of each assay's suitability for use in the laboratories. To enable comparison between assays, an arbitrary scoring system was used to rate specified assay characteristics. Ethical approval was obtained from Muhimbili University of Health and Allied Science Senate Research and Publication Committee. Permission for blood collection was granted by Muhimbili National Hospital and NBTS administration.

## Results

Total of 600 blood samples were included during the study, (130 from VCT clients, 130 from ANC attendees, 140 from blood donors and 200 from CTC patients). Among the 600 samples, 70% (421) of the samples came from females, Majority of the participants aged between 20 to 30 years where as 59%(355) are employed and 71%(424) are married.

Among 600 serum samples that were included in the study, 4 samples gave indeterminate results on immunoblot assay (1 positive from ANC attendee and 3 negative from one blood donor and two ANC attendees) and were excluded in the analysis. A total of 209/596 (35.1%) serum samples were confirmed HIV positive. Of these, the positivity of HIV infection was 2.3% (3/130), 2.3% (3/127), 2.2% (3/139) and 100% (200/200) for VCT clients, ANC attendees, blood donors and CTC patients, respectively. Three hundred and eighty seven (64.9%) were HIV negative samples. The sensitivity of each assay evaluated was 100% (95% CI; 98.3-100%). All reactive samples were concordantly positive among all the three assays.

Initial and repeat specificity of individual HIV ELISAs are summarized in Table 1. The Enzygnost HIV Integral II and Murex HIV Ag/Ab had specificity of 100% (95% CI; 99.1-100%) followed by Vironostika assays. Initially, Vironostika yielded a specificity of 99.2% (Table 2). Three samples were false positive on Vironostika Ag/Ab ELISA. On repeat testing specificity of Vironostika increased to 99.5%.

**Table 1:** Specificity of HIV ELISAs at initial and repeat testing

Assay	Specificity (n = 387)			
	Initial testing		Repeat testing	
	Non reactive	%(95%CI)	Non reactive	%(95%CI)
Enzygnost HIV Integral II	387	100(99.1-100)	-	-
Murex HIV Ag/Ab	387	100(99.1-100)		
Vironostika HIV Uniform II Ag/Ab	384	99.2(97.4-99.7)	385	99.5(98.2-99.9)

**Table 2:** Results on HIV-1 Seroconversion panel AU PRB945

Panel member ID #	Days since 1^st^ bleed	Specimen absorbance/ cut off ratio			Band pattern
		Enzygnost	Murex	Vironostika	Inno-Lia
PRB945-01	0	0.6	0.4		No bands
PRB945-02	3	0.7	0.5	0.5	No bands
PRB945-03	7	1.2	0.7	0.6	No bands
PRB945-04	13	10.7	6.3	0.9	No bands
PRB945-05	15	16.7	13.6	7.9	Indeterminate
PRB945-06	20	20.7	15	13.7	Gp41, p24

Ratios ≥ 1.0 are considered reactive

The mean (optical density (OD)/cut-off (CO)) ratios of samples which were confirmed to be HIV positive and of those which were false positive are summarized in Table 3. The mean OD/CO values of HIV positive samples on all the combination assays were above 10. Samples which gave false positive reactions on Vironostika had lower mean OD/CO values compared to the HIV positive samples.

**Table 3:** Distribution of reactivity's Optical density (OD)/Cut off (CO) ratio by assay

Assay	Confirmed HIV positive			HIV false positives		
	N	Mean (range)	95% CI	n	Mean (range)	95% CI
Enzygnost HIV Integral II	209	13.7 (5.3-31.0)	13.1-14.5	-	-	-
Murex HIV Ag-Ab	209	12.2 (5.2-16.0)	12.0-12.4	-	-	-
Vironostika HIVUniform II Ag-Ab	209	15.5 (12.1-9.0)	15.4-15.7	3	5.7 (1.3-16.5)	-1.6-12.80

The positive predictive value (PPV) for the Enzygnost HIV Integral II and Murex HIV Ag-Ab were 100% (95%CI; 98.3- 100%) and 98.5% (95%CI; 95.28-99.49%) for Vironostika HIV Uniform II Ag-Ab. After repeat testing PPV for Vironostika HIV Uniform II was 99% (95%CI; 96.6-99.9%). The negative predictive value (NPV) was 100% (95%CI; 99.06-100) for all the assays.

Results of testing of a seroconversion panel are summarized in Table 2. Enzygnost HIV Integral II detected HIV infection seven days since first bleed whereas Murex detected first bleed in day 13 and Vironostika detected first bleed in day 15. The 15 and 20 days samples were reactive on all three Ag/Ab combination assays. The specificity of various combinations of ELISAs in HIV testing is shown in Table 4. Initial testing using Murex antigen/antibody assay or Vironostika Antigen/antibody assay followed by testing of reactive samples on the Enzygnost Antigen/antibody assay gave specificity of 100%. Initial testing by Vironostika Antigen/antibody assay followed by Murex Antigen/antibody assay gave 100% specificity.

**Table 4:** Specificity of combinations of ELISAs in HIV testing

Assay	Specificity (n = 387)	
	Non reactive	% (95% CI)
Murex Ag/Ab + Enzygnost HIV Integral II	387	100 (99.1-100)
Vironostika Ag/Ab + Enzygnost HIV Integral II	387	100 (99.1-100)
Vironostika Ag/Ab + Murex Ag/Ab	387	100 (99.1-100)
Enzygnost HIV Integral II+ Murex Ag/Ab	387	100 (99.1-100)
Enzygnost HIV Integral II+ Vironostika Ag/Ab	385	99.5 (98.2-99.9)
Murex Ag/Ab+ Vironostika Ag/Ab	385	99.5 (98.2-99.9)

All the three antigen/antibody combination HIV ELISA were evaluated for operational aspects. Storage conditions (2-8°C); incubation temperature (37°C) and need of special equipment were all the same for all the three Ag/Ab combination assays. Both Enzygnost HIV Integral II and Vironostika carry 1-92 sera per run.

## Discussion

It is important to evaluate performance of HIV Antigen/antibody combination based HIV ELISA continuously to ensure the correct diagnosis of HIV infection especially when the new HIV ELISAs have been developed and introduced in the market. The fourth generation HIV ELISAs are made in a way that HIV p24 antigen is combined with anti-HIV-1 and anti-HIV-2 for early diagnosis of HIV infection. Enzygnost HIV Integral II is among the fourth generation ELISA that was introduced in Tanzania in 2009. In Tanzania ELISA methods are used in Zonal and reference laboratories, National Blood Transfusion Centre and research institutions. The present evaluation determined the performance characteristic of the three HIV Antigen/antibody ELISA.

The study findings showed that all the three combination ELISAs had 100% sensitivity. A previous study conducted in Germany to evaluate the Enzygnost Integral showed 100% sensitivity for the detection of antibodies to HIV-1, groups M and O, and HIV-2 [10]. The antigen /antibody ELISA are designed to be highly sensitive to enable detection of HIV infection as this will provide appropriate and accurate results that will enable distinction between people with infection and those without infection in a health care setup. In another evaluation conducted in UK using five cases detectable only by 4th generation assays, Murex Ag/Ab exhibited sensitivity of 100% which is in agreement with the finding of the present study [21].

In the present study, Enzygnost exhibited the specificity of 100% which is in contrast to the findings from the previous evaluation done in Germany using 4002 unselected blood donors, where the specificity of Enzygnost Integral II was found to be 99.78% initially and 99.80% after retesting [10]. Based on evaluation of 388 samples, the specificity for Murex Ag-Ab combination assay (100%) was higher as compared to a specificity of 99.4% on a previous study conducted in Tanzania in 2006 [8]. In another evaluation which used 9,289 samples [14], Murex showed a specificity of 99.7% which is lower than the specificity in the present study. The specificity of the Vironostika was found to be lower (99.2% at initial testing and 99.5% at repeat testing) compare to the specificity of Enzygnost Integral II and Murex. In another evaluation, the specificity for the Vironostika was 98.9% at initial testing and 99.4% at repeat testing [8] which is lower as compared to the present study. One serum that gave negative result after repeat testing increased specificity of Vironostika Ag-Ab to 99.5%. There is a possibility that the initial false positive results obtained when testing these specimens on Vironostika was due to technical error. After repeat testing on the Vironostika, specificity was still lower compared to the Enzygnost HIV Integral II and Murex Ag-Ab; both of the lower specificities were contributed to the false positives results, the false positive results may have been contributed by cross reactions in the sera. This is similar to the previous study conducted in Tanzania [8].

In the present study, samples that were confirmed HIV positive had high mean OD: CO ratio on all the three assays (13.7, 12.2 and 15.2 in Enzygnost, Murex and Vironostika respectively) compared to samples that gave false positive results reaction on Vironostika assays (5.7). Recent studies have shown that samples which are repeatedly reactive in sequential antibody screening assays but which are WB negative should be interpreted with caution regarding their HIV serostatus because some HIV-1 antibody assays have been found to be reactive earlier in the infection process than WB [22, 23]. It is therefore important to take special precautions in those samples showing low OD: CO values

In the present study, the Enzygnost HIV Integral II assay detected HIV infection seven days since first bleed, compared to the Murex and Vironostika Ag-Ab ELISAs which were able to detect infection on thirteenth day after infection. This is similar to comparative evaluations of the fourth generation HIV screening assays performed on the seroconversion panels which have shown that the fourth generation HIV screening assays reduced the diagnostic window of HIV infection by an average of seven days and that they permit an earlier diagnosis of HIV infection by detecting p24 antigen which may be present in samples from individuals with recent HIV infection prior to seroconversion [10, 12–14, 17, 18, 24–27]. It is important to utilize Antigen/antibody combination based ELISA especially in public health arena, where secondary prevention programs involve early disease detection and intervention. Results from the present study indicated that detection is 7–15 days earlier with the new combined antigen/antibody HIV ELISA, as this will prevent transfusing infected blood from donated blood and also will reduce infections from mother to child. The gold standard that was used could not detect any bands early during seroconversion because it has limited ability to detect antibodies during early HIV infection. Western blot assay can detect antibody at least 3 weeks from the initial HIV infection.

It is necessary to utilize a combination of HIV antigen-antibody assays of HIV infection before transfusion and for diagnosis of HIV in individuals suspected of infection [26]. Even if screening will involve combination of Ag-Ab assays, there is still a need to have a test that will resolve the discordant results because the two assays can lead to false positive or false negative results [8, 26, 28]. Although both Murex and Enzygnost had 100% sensitivity, 100% specificity and 100% PPV of 100%, the study suggests Murex to be used as first screening assay followed by testing reactive samples on Enzygnost due to high cost of Enzygnost. Lower specificity of Vironostika suggests either screening with Vironostika followed by testing reactive samples on Enzygnost or Murex. Samples giving repeatedly discordant results on the Ag/Ab combination assays to be tested with Western blot, however for resource limited country like Tanzania, use of western blot are very costly; hence it is suggested to collect another sample for retesting.

Performance characteristic of the assays for the specific categories of the study participants showed that a combination comprising of Vironostika as a first test followed by Murex or Enzygnost as a second test would be suitable for VCT, Blood donors, ANC and HIV/AIDS patients taking into account the element of cost. All the assays have sensitivity of 100%, any of the three can be used as the first assay although Enzygnost HIV Integral II is very expensive compared to Vironostika and Murex

All the kits have same operational characteristic in terms of Storage conditions (2-8°C), incubation temperature (37°C) and need of special equipment. When comparing the cost of the kits per these assays, Murex and Vironostika are preferable to Enzygnost as Enzygnost is expensive compare to Murex and Vironostika, however, both Enzygnost HIV Integral II and Vironostika carry 1-92 sera per run while Murex carry 1-90 sera per run. Performance appraisal revealed highest score for easiness for all the assays; however duration of time of testing and number of steps for Enzygnost HIV Integral II were reported to be longer compared to the other two assays evaluated.

Based on performance characteristics, all the assays qualified for inclusion in the National algorithm. Seroconversion results revealed better performance for Enzygnost as it detected HIV infection seven days earlier than Murex and Vironostika; however operational characteristics support Murex and Vironostika to be preferable to Enzygnost.

## Conclusion

Initial testing using either Vironostika or Murex HIV antigen/antibody combination ELISA followed by testing of reactive samples on the Enzygnost HIV Integral II gave sensitivity, specificity, PPV and NPV of 100% with reduced window period. Based on the current evaluation all the assays have good performance characteristics and all the assays qualified for inclusion in the National algorithm. Regarding on operational characteristics, an element of cost was considered when recommending for the algorithms to be used. The recommended algorithms are Vironostika Ag/Ab as initial testing followed by testing reactive samples on Murex Ag/Ab; Vironostika Ag/Ab as initial testing followed by testing reactive samples on Enzygnost HIV Integral II; and Murex as initial testing followed by testing reactive samples on Enzygnost HIV Integral II.

## References

[1] Constantine NT (1993). Serologic tests for the retroviruses: approaching a decade of evolution. Aids..

[2] Poljak M (2002). Retrospective evaluation of the Vidas HIV DUO test for simultaneous detection of anti-HIV antibody and p24 antigen, Acta dermatovenerologica Alpina. Panonica et Adriatica..

[3] UNAIDS/WHO, Joint United Nations Programme on HIV/ AIDS (1997). Revised recommendations for the selection and use of HIV antibody tests.

[4] Urassa WK (1992). Alternative confirmatory strategies in HIV-1 antibody testing. JAIDS Journal of Acquired Immune Deficiency Syndromes..

[5] Carvalho MB, Vaz RS FOJ (1996). Risk factor analysis and serological diagnosis of HIV-1/HIV-2 infection in a Brazilian blood donor population: validation of the World Health Organization strategy for HIV testing. AIDS..

[6] Meda N, Soudré RB, Dahourou H, Ouedraogo-Traoré R, Ouangré A, Bambara A, Kpozehouen A, Sanou H, Valéa D, Ky F, Cartoux M, Barin FV, de PP (1999). Serological diagnosis of human immuno-deficiency virus in Burkina Faso: reliable, practical strategies using less expensive commercial test kits. Bull World Health Organization..

[7] Andersson S, Norrgren H, Dias F (1997). Field evaluation of alternative testing strategies for diagnosis and differentiation of HIV-1 and HIV-2 infections in an HIV-1 and HIV-2-prevalent area. AIDS..

[8] Aboud S, Lyamuya E, Mhalu F, Biberfeld G (2006). Evaluation of HIV antibody and antigen/antibody combination ELISAs for use in an alternative confirmatory HIV testing strategy in Dar es Salaam, Tanzania. Journal of virological methods..

[9] Yerly S (1999). Early diagnosis of primary HIV infections: using a combined screening test (p24 antigen and anti-HIV antibodies. Schweizerische medizinische Wochenschrift..

[10] Brust S (2000). Shortening of the diagnostic window with a new combined HIV p24 antigen and anti-HIV-1/2/O screening test. Journal of virological methods..

[11] Couroucé AM, Maniez M, Janot C, Noel L, Elghouzzi MH (1992). Effectiveness of assays for antibodies to HIV and p24 antigen to detect very recent HIV infection in blood donors. AIDS..

[12] Gürtler L, Michl U, Hofmann H, Paggi GG, Bossi V, Thorstensson R, Villaescusa RG, Eiras A, Hernandez JH, Melchior W, Donié F, Weber B (1998). Reduction of the diagnostic window with a new combined p24 antigen and human immunodeficiency virus antibody screening assay. Virology methods..

[13] Laperche S, Courouce AM (2000). Screening tests combined with p24 antigen and anti-HIV antibodies in early detection of HIV-1 Transfusion. Clinical biology..

[14] Ly TD (2001). Seven human immunodeficiency virus (HIV) antigen-antibody combination assays: evaluation of HIV seroconversion sensitivity and subtype detection. Journal of clinical microbiology..

[15] Ly TD (2001). Early detection of human immunodeficiency virus infection using third- and fourth-generation screening assays. Clinical microbiology of infectious diseases..

[16] Saville RD, Jack N (2001). Fourth-generation enzyme-linked immunosorbent assay for the simultaneous detection of human immunodeficiency virus antigen and antibody. Clinical microbiology of infectious diseases..

[17] Van Binsbergen J, Jacobs Keur W, Bruynis F, van de Graaf M, van der Heijden J, Kambel JT D (1999). Improved performance in seroconversion with a 4th generation HIV antigen/antibody assay. Virology methods..

[18] Weber B, Berger A, Doerr HW (1998). Reduction of diagnostic window by new fourth-generation human immunodeficiency virus screening assays. Clinical microbiology of infectious diseases..

[19] Ly TD, Brennan C, Vallari A, Ebel A, Hunt J (2004). Evaluation of the sensitivity and specificity of six HIV combined p24 antigen and antibody assays. Virology methods..

[20] Weber B, Rabenau H, Doerr HW (2002). Evaluation of a new combined antigen and antibody human immunodeficiency virus screening assay. VIDAS HIV DUO Ultra. Clinical microbiology of infectious diseases..

[21] McElborough D (2004). Importance of using an HIV Ag/Ab combined assay in a UK population at high risk of acquiring HIV infection. Communicable diseases public health..

[22] Tamashiro H, Maskill W, Emmanuel J, Fauquex A, Sato P (1993). Reducing the cost of HIV antibody testing. Lancet..

[23] Zaaijer HL (1992). Early detection of antibodies to HIV-1 by third-generation assays. The Lancet..

[24] Ly TD, Laperche S, Faucher V, Ebel A, Seine I, Ag HI V (2005). Evaluation of three New Combined HIV p24 Antigen and Antibody Assays. J Virol Methods.

[25] O'Brien TR, Epstein JS, Holmberg SD, Schochetman G (1992). Testing for antibodies to human immunodeficiency virus type 2 in the United States: Morbidity and mortality weekly report Recommendations and reports/Centers for Disease Control..

[26] Urassa W (1999). The accuracy of an alternative confirmatory strategy for detection of antibodies to HIV-1: experience from a regional laboratory in Kagera, Tanzania. Journal of clinical virology..

[27] Van Binsbergen J, Keur W, Siebelink A, van de Graaf FM, Jacobs A, de Rijk D, Nijholt L, Toonen J, Gürtler LG (1998). Strongly enhanced sensitivity of a direct anti-HIV-1/-2 assay in seroconversion by incorporation of HIV p24 Ag detection: a new generation Vironostika HIV Uni-Form II. Virology methods..

[28] Urassa W (2002). Evaluation of an alternative confirmatory strategy for the diagnosis of HIV infection in Dar Es Salaam, Tanzania, based on simple rapid assays. Journal of virological methods..

